# Endometrial gene expression reveals compromised progesterone signaling in women refractory to embryo implantation

**DOI:** 10.1186/1477-7827-12-92

**Published:** 2014-09-23

**Authors:** Alejandro Tapia-Pizarro, Paula Figueroa, Julio Brito, Juan Carlos Marín, David J Munroe, Horacio B Croxatto

**Affiliations:** Instituto de Investigaciones Materno Infantil, Facultad de Medicina, Universidad de Chile, Santiago, Chile; Facultad de Medicina, Universidad Mayor, Santiago, Chile; Facultad de Ciencias, Universidad del Bio-Bío, Chillán, Chile; Advanced Technology Program, SAIC-Frederick, Inc., National Cancer Institute-Frederick, Frederick, MD 21701 USA; Center for Integrative Medicine and Innovative Sciences, Universidad Andrés Bello, Santiago, Chile

**Keywords:** Endometrium, Gene expression, PROGINS, Implantation failure

## Abstract

**Background:**

Endometrial function is essential for embryo implantation. The aim of this study was to analyze gene expression profiles from individual endometrial samples obtained from women with repeated implantation failure after IVF in oocyte donation programs.

**Methods:**

Seventeen volunteers were recruited: women who had previously participated as recipients in oocyte donation cycles and repeatedly exhibited implantation failure (Group A, study group, n = 5) or had at least one successful cycle (Group B, control group, n = 6) and spontaneously fertile women (Group C, normal fertility group, n = 6). An endometrial cycle was induced with exogenous estradiol (E2) and progesterone (P) and an endometrial sample was collected on the seventh day of P treatment.

**Results:**

Transcriptome analysis showed 82 genes with consistent differential gene expression when comparing A vs. B and A vs. C. One hundred transcripts differentially expressed in group A vs. B have been shown to be regulated by P, suggesting compromised P signaling in the endometrium. The P receptor (PR) mutation *PROGINS* was not detected in women from group A. Semi-quantitation of immunoreactive PRA/B, PRB and Sp1 (a transcription factor related to P signaling) in paraffin-embedded endometrial sections, did not show statistically significant differences amongst groups. However immunostaining glycodelin was significantly decreased in endometrial samples from group A

**Conclusions:**

We conclude that some cases of repeated implantation failure could be associated with an aberrant gene expression profile. Compromised P signaling might be the underlying mechanism for such endometrial gene expression deregulation in women with repeated implantation failure.

## Background

Cellular and molecular events in the uterine milieu that lead to successful blastocyst implantation are required in the endometrium to become receptive and ready for implantation. Acquisition of receptivity is driven by estradiol (E2) and progesterone (P), which acting through their receptors, change the transcription rate of target genes [1]. Particularly, the postovulatory rise in P triggers a sequence of highly coordinated responses beginning with the detention of the estrogen-induced epithelial cell proliferation and followed by the transformation to a secretory phenotype of the gland, recruitment of leukocytes and angiogenesis [2]. The P action is mediated primarily through binding to and activation of its cognate receptors; the full length B- and N-terminally truncated A isoforms of the P receptor (PR), classically defined as ligand-activated transcription factors [3]. Upon exposure to P, the ligand-activated receptor can directly interact with specific P-response elements (PREs) in the promoter regions of target genes. It is accepted that P acts on an estrogen primed endometrium to initiate a pattern of gene expression important for achievement of receptivity and an altered PR signaling has been associated with human endometrial dysfunction [4]. The antiprogestin mifepristone binds to the PR with high affinity blocking the biological effects of P. In women, the administration of a single dose of oral mifepristone (200 mg) during the secretory phase of the cycle rapidly renders the endometrium unreceptive and modifies gene expression in the uterus within 6 h of administration [5–7].

The development of microarray technology has led to many large-scale gene expression profiling studies of human endometrium [8–10]. Although there seems to be very few consensus genes that have been identified across similar studies [11], collectively they demonstrate that a multitude of genes are associated with the endometrial transcriptome, whose regulation for the acquisition of the receptive phenotype is ultimately driven by P. The approach our group has used to identify the endometrial receptivity transcript profile in a previous report from our laboratory [12] was to analyze endometrial tissue obtained from women during a mock hormonal treatment cycle for oocyte donation as a recipient. The endometrial samples are collected during the time interval corresponding to the window of implantation [13] comparing gene expression profiles from women who were refractory to implantation and those who achieved pregnancy in previous oocyte donation cycles [12]. Although this previous study provided interesting insights to endometrial gene expression associated with implantation failure, the microarrays analysis was performed with only 3(from a total of 5) samples from women with repeated embryo implantation failure that were pooled and using a microarrays platform that examined only one-third of the human genome; providing only a partial view of the whole picture.

The aim of the present report was to examine the individual gene expression profiles in the endometrium from women with implantation failure and compare them with those obtained from fertile women in order to identify compromised transcripts and pathways in the infertile group. We used a microarrays platform for complete coverage of the human genome and bioinformatics tools for data interpretation. Here we report that several transcripts, whose expression level is aberrant in the infertile group, have been described as regulated by P and are related to immune function.

## Methods

### Subjects

This study was approved by the Ethics Review Committee for Investigations in Human Beings of Faculty of Medicine, University of Chile: protocol No. 093–2008, approved 12-29-2008, initiated 01-05-2009 finished 03-31-2014. Each volunteer participating read and signed the informed consent approved by the respective Ethics Review Committee.

Three groups of women were recruited as has been described elsewhere [12]. Group A (n = 5) consisted of women that had never been pregnant and had previously participated in two or more cycles as recipients in an oocyte donation program with no evidence of embryo implantation. Male partners had normal seminal parameters and transferred embryos had good morphology, at least equivalent to embryos transferred to the oocyte donor who became pregnant. Since good quality embryos with the ability to implant and develop normally derive from good quality oocytes, it was required that the oocyte donor had become pregnant from the same oocyte pool. Women from group A were recruited within 3 years following the last failed cycle. Group B (n = 6) comprised of women who became pregnant as recipients in previous oocyte donation cycles and delivered live infants. Group C (n = 6) included normal fertile women who conceived in natural cycles and had three or more live births and had elective tubal ligation at least 1 year prior to their participation for reasons unrelated to this study. Women from groups B and C were recruited within 5 years following the last successful pregnancy. The general exclusion criteria for all volunteers included: metabolic or endocrine diseases other than those leading to ovarian failure, chronic use of medication other than HRT, polycystic ovary syndrome, drug abuse, obesity, endometriosis, pelvic inflammatory disease and current genital tract infection. Age and body mass index from recruited women as well as the plasma P and endometrial thickness measured on the day of the endometrial collection are shown in Table 1.

**Table 1:** **Characteristics of women participating in the study and parameters evaluated during the hormonal replacement cycle**

	Group A (n = 5)	Group B (n = 6)	Group C (n = 6)	P value
*Age (years)*	35.4 (26–43)	41.9 (34–46)	41.3 (36–47)	0.1117
*Body mass index*	25.2 (22.6-29.4)	25.6 (22.5-27.4)	25.4 (23.3-28.1)	0.573
Plasma progesterone* (nmol/L)	75.7 (38–122)	88.1 (36–192)	63.7 (43.3-75)	0.7601
Endometrial thickness* (mm)	10.8 (9–12)	9.1 (8–10)	11.2 (8.5-12.5)	0.6162

*On the day of endometrial collection.

### Induction of endometrial cycle

All subjects underwent the induction of an artificial endometrial cycle with exogenous ethinyl E2 for 20 days and for the last 7 days, this treatment was administered concomitantly with micronized P as described previously [12]. An endometrial sample was obtained on 20th day of the endometrial cycle. One part of the specimen was snap frozen in liquid nitrogen and kept at -80 ° C until use and the remaining portion was fixed in paraformaldehyde for histological dating, according to the criteria of Noyes *et al.*, [14] and for immunohistochemistry (IHC) studies. All biopsies were classified as normal secretory endometrium with no signs of inflammatory processes.

### Gene expression profiling

Total RNA was extracted from frozen endometrial tissue samples using Trizol (Invitrogen, Gaithersburg, MD, USA) as directed by the manufacturer and then checked for yield and quality as described before [12]. The Human Genome U133 plus 2.0 GeneChip oligonucleotide microarrays (Affymetrix, Sunnyvale, CA, USA); corresponding to 47,000 transcripts and variants, including 38,500 well-characterized human genes, was used for gene expression analysis according to the manufacturer’s instruction.

Microarrays Data Analysis: Replicate hybridizations were performed for each RNA sample and raw data obtained from the GeneChip Microarray Suite v 1.4 was subsequently analyzed using the National Cancer Institute’s Microarrays Data Base webtool (mAdb) (http://nciarray.nci.nih.gov).

### Statistical analyses of microarrays data

Significant genes were defined as ≥2, *p*-value < 0.001 and a false discovery rate (FDR) < 0.1 [15]. *T*-test was performed to determine statistical differences and from the significant genes identified, Venn diagrams were constructed to identify coincident transcripts.

#### Hierarchical clustering

Was performed based on uncentered correlations with average linkage clustering using mAdb. The resulting dendogram allows data structure visualization of endometrial samples according to total gene expression, revealing samples with similar patterns of gene expression and relationships between the specimens.

#### Principal component analysis (PCA)

Was performed for simplifying the large amount of data derived from microarray analysis [16]. We applied the unbiased PCA algorithm to all samples using all transcripts analyzed with the microarray chip to look for expression patterns and underlying cluster structures of endometrial samples.

#### Functional clustering

To increase the effectiveness of DNA microarray analysis, data sets of differentially expressed genes from the comparison between A vs. B and A vs. C were intersected to define those transcripts consistently up- or down-regulated and combined with external data sources, such as gene annotation, in order to associate the expression patterns of this particular set of genes with the biological processes that they may represent. In our analysis, we submitted our gene lists to the web-based tools DAVID (Database for Annotation, Visualization and Integrated Discovery) [17] and GATHER (Gene Annotation Tool to Help Explain Relationships) [18] for functional annotation analysis in order to gain an in-depth understanding of their biological themes, which otherwise would require laborious and somewhat subjective manual literature searches.

### DAVID

Up- and down-regulated genes were submitted to DAVID database for systematically extracting biological meaning for them by retrieving pathway maps from the Kyoto Encyclopedia of Genes and Genomes (KEGG) [19] and Biocarta pathways database (http://www.biocarta.com/genes/index.asp) along with Gene Ontology (GO) functional annotations from Entrez Gene [20]. The parameters of the “Functional Annotation Clustering” (a part of the “Functional Annotation Tool”) were set to the highest level of stringency in order to obtain the smallest number of maps. The DAVID database associates each annotation to a gene group using a contingency table representation and calculates its significance.

### GATHER

Regulated genes were submitted as well to GATHER database (http://gather.genome.duke.edu/) that contains the GO annotations and KEGG pathways. The GATHER database associates each group of transcripts with the same functional annotation and calculates a Bayes factor [18] which is a measure of the strength of the evidence supporting an association of an annotation with the submitted gene list. We have selected a low Bayes factor (≥3) for presenting the preponderant evidences for associations.

#### Immunohistochemistry (IHC)

A portion of each endometrial sample was fixed in paraformaldehyde, included in paraffin blocks and 5 μm sections were prepared. PR-A/B, PR-B, glycodelin and Specificity protein 1 (Sp1) were evaluated by IHC in the endometrial samples using the antibodies and dilutions shown in Table 2 and the broad spectrum Histostain-SP kit (Life Technologies, Carlsbad, CA, USA) as described previously [21]. Immunoreactive PRA/B, PRB, Sp1 and glycodelin in endometrial sections was semi-quantified using the expression level score (ELS), calculated by means of Image Pro Plus software (Media Cybernetics Rockville, MD, USA) as described previously [21]. Briefly, ELS = Mean Optical Density of immunostaining x Percent Area Positively Stained x 100.

**Table 2:** **Antibodies and dilutions used for immunohistochemistry**

Antibody	Source	Dilution
Progesterone receptor (PR)-A/B	Santa Cruz Biotech. (sc-810)	1:50
PR-B	Novocastra (NCL-PGR-B)	1:100
Glycodelin	H. Koistinen [22]	1:1000
Specificity protein-1 (Sp1)	Santa Cruz Biotech. (sc-14027)	1:100

#### DNA isolation and *PROGINS*detection

Genomic DNA was isolated from leukocytes derived from peripheral blood obtained by venipuncture using the PAXgene Blood DNA Validation kit (Qiagen, Valencia, CA, USA) following the manufacturer’s protocol. The detection of Alu insertion in intron G and restriction fragment length polymorphism (RFLP) analysis in exon 5 to confirm the presence of *PROGINS* mutation was performed as described by Pisarska *et al.* [23].

## Results

### Gene expression profile analysis

**Figure 1:**
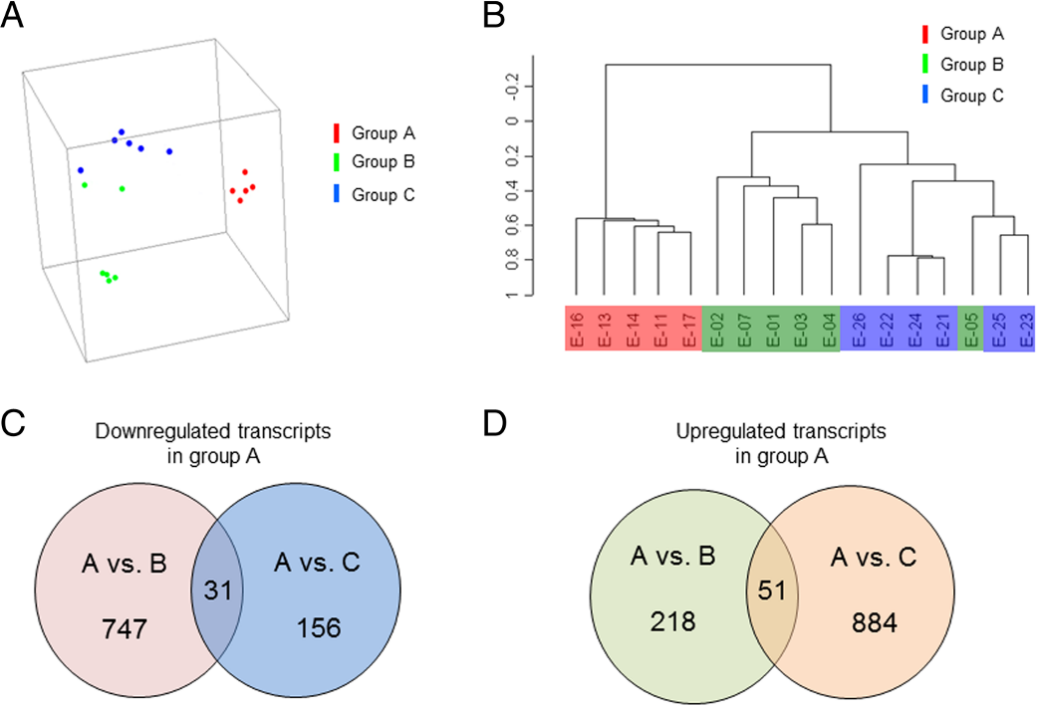
**Principal component analysis (PCA) plot of gene expression profiles from endometrial samples and Venn diagrams from differentially expressed transcripts. A**, The profiles from infertile women (group A (n = 5); red dots) cluster separately to clusters representative of women with embryo implantation (groups B (n = 6) and C (n = 6); green and blue dots, respectively). **B**, hierarchical clustering analysis represented in a tree-like dendogram revealing the similarities on gene expression profiles of endometrial samples. A clear segregation of samples into two major clustering branches, one with samples from group A and the other with samples form groups B and C that self-cluster together. **C**, Venn diagrams showing the differentially expressed genes in each group, which are either 2-fold down- (panel **C**) or up-regulated (panel **D**) in endometrial samples from women with implantation failure (group A) compared to those from women that conceived either by IVF (Group B) or naturally (group C).

Women with implantation failure (group A, n = 5), women with 2 or more livebirths conceived either by oocyte donation (group B, n = 6) or naturally (group C, n = 6) were subjected to an oocyte donation mock cycle as recipients and on the seventh day of P administration an endometrial sample was obtained. Total RNA was extracted from each tissue sample and used to individually probe the HG_U133 plus 2.0 human gene microarray comprising of 54,675 genes and expressed sequence tags.We performed PCA for all the endometrial samples analyzed using their respective gene expression profiles for their representation on a three-dimensional graphic (Figure 1A). Each point in a PCA graph represents the gene expression profile of an endometrial sample and the distance between two plotted points is proportional to the degree of similarity between the gene expression profiles. The PCA plot comprising of a projection on the first three principal components, which together explain 48.8% (21%, 14%, and 13%) of the total variance, showed that endometrial samples from infertile subjects clustered apart from samples belonging to the control groups. In addition, gene expression profiles from endometrial samples obtained from microarray analysis were subjected to unsupervised hierarchical clustering analysis in order to generate a dendogram, which is a tree-structured graph that illustrates the similarities in gene expression profiles between endometrial samples from all groups. The dendogram obtained displayed a striking segregation of samples into two major clustering branches, corresponding to the implantation failure group (Group A) and the successful implantation groups (Groups B and C, Figure 1B).

The microarrays data analysis revealed that 747 transcripts were down-regulated in group A compared to group B; whereas 218 transcripts were up-regulated (Figure 1C and 1D). When group A was compared to group C, 156 and 884 transcripts were decreased and increased respectively in group A (Figure 1C and 1D). Only 31 and 51 transcripts down- and up-regulated respectively were common when comparing group A with the control groups B and C (Figure 1C and 1D). The lists of common differentially expressed genes in the comparison of gene expression profiles from group A vs. Group B and Group A vs. group C is in Table 3 for down- and Table 4 for up-regulated transcripts. An independent validation by Real Time RT-PCR for some of the differentially expressed genes found in the samples used in this study has been reported elsewhere [12], supporting our microarrays findings.

**Table 3:** **Genes whose transcript level was down-regulated in Group A (n = 5) when compared with Group B (n = 6) and Group C (n = 6) in the microarray analyses**

UniGene ID	Gene symbol	Gene title	A vs. B	p value	A vs. C	p value	Average
Hs.699841	IGHA1	Immunoglobulin heavy constant alpha 1	0.1	0.00086	0.08	0.00043	0.09
Hs.436657	CLU	Clusterin	0.11	0.00043	0.17	0.00013	0.14
Hs.356624	NID1	Nidogen 1	0.19	0.00013	0.14	0.00022	0.16
Hs.82071	CITED2	Cbp/p300-interacting transactivator. with Glu/Asp-rich carboxy-terminal domain. 2	0.15	0.00086	0.19	0.00043	0.17
Hs.532325	PAEP	Progestagen-associated endometrial protein (PAEP)	0.09	0.00086	0.29	0.00086	0.19
Hs.38972	TSPAN1	Tetraspanin 1	0.14	0.00022	0.24	0.00086	0.19
Hs.445705	RRM1	Ribonucleotide reductase M1	0.18	0.00043	0.23	0.00022	0.2
Hs.1012	C4BPA	Complement component 4 binding protein. alpha	0.21	0.00043	0.22	0.00043	0.22
Hs.513261	HN1L	Hematological and neurological expressed 1-like	0.18	0.00022	0.35	0.00043	0.26
Hs.80658	UCP2	Uncoupling protein 2 (mitochondrial. proton carrier)	0.1	0.00043	0.49	0.00013	0.29
Hs.502989	UNC93B1	Unc-93 homolog B1 (C. elegans)	0.23	0.00013	0.37	0.00043	0.3
Hs.414099	CNPY3	Canopy 3 homolog (zebrafish)	0.29	0.00013	0.32	0.00022	0.31
Hs.110571	GADD45B	Growth arrest and DNA-damage-inducible. beta	0.2	0.00022	0.44	0.00086	0.32
Hs.320151	AGPAT2	1-acylglycerol-3-phosphate O-acyltransferase 2 (lysophosphatidic acid acyltransferase. beta)	0.23	0.00086	0.43	0.00013	0.33
Hs.77422	PLP2	Proteolipid protein 2 (colonic epithelium-enriched)	0.26	0.00043	0.42	0.00043	0.34
Hs.1497	RARG	Retinoic acid receptor. gamma	0.33	0.00043	0.35	0.00043	0.34
Hs.389700	MGST1	Glutathione S-transferase. microsomal	0.4	0.00022	0.29	0.00043	0.34
Hs.292078	LARP1	La ribonucleoprotein domain family. member 1	0.34	0.00043	0.36	0.00086	0.35
Hs.334587	RBPMS	RNA binding protein with multiple splicing	0.29	0.00086	0.43	0.00022	0.36
Hs.5298	ADIPOR1	Adiponectin receptor 1	0.3	0.00022	0.42	0.00013	0.36
Hs.439894	CASZ1	Castor zinc finger 1	0.24	0.00022	0.49	0.00086	0.37
Hs.371727	SCNN1G	Sodium channel. nonvoltage-gated 1. gamma	0.29	0.00013	0.45	0.00043	0.37
Hs.474596	LIMK2	LIM domain kinase 2	0.26	0.00043	0.5	0.00013	0.38
Hs.459940	LITAF	Lipopolysaccharide-induced TNF factor	0.37	0.00022	0.39	0.00086	0.38
Hs.442449	CHST14	Carbohydrate (N-acetylgalactosamine 4–0) sulfotransferase 14	0.42	0.00086	0.35	0.00086	0.38
Hs.518525	GLUL	Glutamate-ammonia ligase	0.42	0.00043	0.48	0.00086	0.45
Hs.119177	ARF3	ADP-ribosylation factor 3	0.47	0.00013	0.45	0.00043	0.46
Hs.497417	KIAA0317	KIAA0317	0.49	0.00086	0.44	0.00013	0.46
Hs.501728	RHOG	Ras homolog gene family. member G (rho G)	0.46	0.00043	0.47	0.00043	0.47
Hs.414614	SCNN1B	Sodium channel. nonvoltage-gated 1. beta	0.48	0.00013	0.47	0.00022	0.47
Hs.436896	POLR3A	Polymerase (RNA) III (DNA directed) polypeptide A. 155 kDa	0.48	0.00043	0.47	0.00043	0.48

Data includes genes with decreased transcript levels displaying a ≥2-fold difference in average A vs. B and A vs. C.

**Table 4:** **Genes whose transcript level was up-regulated in Group A (n = 5) when compared with Group B (n = 6) and Group C (n = 6) in the microarray analyses**

UniGene ID	Gene symbol	Gene title	A vs. B	p value	A vs. C	p value	Average
Hs.35086	USP1	Ubiquitin specific protease 1 (USP1), mRNA.	42.52	0.00022	44.32	0.00043	43.42
Hs.436977	SYTL3	Synaptotagmin-like 3	20.68	0.00043	26.72	0.00013	23.70
Hs.133421	LIFR	Leukemia inhibitory factor receptor	43.71	0.00086	3.32	0.00022	23.52
Hs.160211	THRAP3	Thyroid hormone receptor associated protein 3 (THRAP3), mRNA.	29.04	0.00013	3.66	0.00043	16.35
Hs.532399	ZC3H11A	KIAA0663 gene product (KIAA0663), mRNA.	4.38	0.00043	28.05	0.00086	16.21
Hs.652169	PLGLB2	Plasminogen-like B2	21.71	0.00086	9.45	0.00086	15.58
Hs.524809	CLIP1	Restin (Reed-Steinberg cell-expressed intermediate filament-associated protein) (RSN), transcript variant 2, mRNA.	2.19	0.00043	25.81	0.00043	14.00
Hs.16355	MYH10	Myosin, heavy polypeptide 10, non-muscle (MYH10), mRNA.	2.6	0.00013	24.08	0.00022	13.34
Hs.502829	SF1	Splicing factor 1 (SF1), transcript variant 4, mRNA.	21.86	0.00043	4.08	0.00086	12.97
Hs.517949	MAP4	Microtubule-associated protein 4 (MAP4), transcript variant 1, mRNA.	10.41	0.00043	11.96	0.00013	11.18
Hs.8118	SMCHD1	KIAA0650 protein	18.77	0.00013	2.17	0.00043	10.47
Hs.514806	GALNT1	UDP-N-acetyl-alpha-D-galactosamine:polypeptide N-acetylgalactosaminyltransferase 1 (GalNAc-T1) (GALNT1), mRNA.	11	0.00043	9.38	0.00043	10.19
Hs.130293	LUC7L3	Cisplatin resistance-associated overexpressed protein (CROP), transcript variant 2, mRNA.	6.32	0.00043	11.88	0.00022	9.10
Hs.143728	WASL	Wiskott-Aldrich syndrome-like (WASL), mRNA.	2.07	0.00022	14.32	0.00086	8.20
Hs.532082	IL6ST	Interleukin 6 signal transducer (gp130, oncostatin M receptor) (IL6ST), transcript variant 2, mRNA.	2.95	0.00013	13	0.00043	7.97
Hs.2913	EPHB3	EphB3 = HEK2 = tyrosine kinase receptor = large erk kinase	11.39	0.00086	4.41	0.00086	7.90
Hs.431081	USP53	Ubiquitin specific protease 53	2.23	0.00013	13.55	0.00043	7.89
Hs.194726	BAG4	BCL2-associated athanogene 4 (BAG4), mRNA.	11.71	0.00013	3.16	0.00086	7.44
Hs.464971	PIK3C3	Phosphoinositide-3-kinase, class 3	3.92	0.00043	10.41	0.00086	7.16
Hs.9997	SECISBP2L	KIAA0256 gene product (KIAA0256), mRNA.	5.43	0.00086	7.36	0.00013	6.39
Hs.497788	EPRS	Glutamyl-prolyl-tRNA synthetase (EPRS), mRNA.	4.41	0.00043	8.11	0.00043	6.26
Hs.101014	CEP57	Translokin (KIAA0092), mRNA.	5.03	0.00086	7.31	0.00086	6.17
Hs.143600	GOLIM4	Golgi phosphoprotein 4 (GOLPH4), mRNA.	2.46	0.00043	9.85	0.00022	6.16
Hs.24485	SMC3	Chondroitin sulfate proteoglycan 6 (bamacan) (CSPG6), mRNA.	2.53	0.00022	9.65	0.00043	6.09
Hs.193832	GPATCH4	G patch domain containing 4 (GPATC4), transcript variant 3, mRNA.	3.32	0.00013	7.94	0.00086	5.63
Hs.406695	PRDM7	PR domain containing 7 (PRDM7), mRNA.	2.04	0.00086	8.46	0.00086	5.25
Hs.42194	SPCS3	Signal peptidase complex subunit 3 homolog (S. cerevisiae) (SPCS3), mRNA.	2.1	0.00043	8.34	0.00022	5.22
Hs.458418	KIAA1731	PREDICTED: KIAA1731 protein (KIAA1731), mRNA.	2.04	0.00013	8.4	0.00086	5.22
Hs.49853	CCAR1	Cell division cycle and apoptosis regulator 1	2.75	0.00086	7.67	0.00013	5.21
Hs.496414	ATP7A	ATPase, Cu++ transporting, alpha polypeptide (Menkes syndrome)	2.89	0.00086	7.16	0.00043	5.02
Hs.481181	NEK1	NIMA (never in mitosis gene a)-related kinase 1 (NEK1), mRNA.	3.07	0.00086	5.35	0.00043	4.21
Hs.440833	PKN2	protein kinase N2 (PKN2), mRNA.	6.02	0.00022	2.14	0.00043	4.08
Hs.524009	AASDHPPT	Aminoadipate-semialdehyde dehydrogenase-phosphopantetheinyl transferase	3.89	0.00013	4.2	0.00086	4.04
Hs.26904	SEC63	SEC63 homolog (S. cerevisiae)	2.95	0.00086	3.78	0.00043	3.37
Hs.93485	SCN2A	MRNA; cDNA DKFZp761D191 (from clone DKFZp761D191)	2.08	0.00043	4.5	0.00013	3.29
Hs.31082	TMEM33	Transmembrane protein 33	2.19	0.00086	4.35	0.00043	3.27
Hs.371372	CWC27	Serologically defined colon cancer antigen 10 (SDCCAG10), mRNA.	2.95	0.00043	3.51	0.00086	3.23
Hs.523299	EIF3A	Eukaryotic translation initiation factor 3, subunit 10 theta, 150/170 kDa (EIF3S10), mRNA.	2.68	0.00022	3.58	0.00022	3.13
Hs.440320	CUL5	Cullin 5 (CUL5), mRNA.	2.6	0.00043	3.25	0.00043	2.93
Hs.203965	PHTF2	Putative homeodomain transcription factor 2	3.56	0.00013	2.3	0.00086	2.93
Hs.335068	TGS1	Nuclear receptor coactivator 6 interacting protein (NCOA6IP), mRNA.	3.63	0.00086	2.16	0.00013	2.89
Hs.189075	TWF1	Twinfilin, actin-binding protein, homolog 1 (Drosophila)	2.36	0.00086	2.97	0.00043	2.67
Hs.127310	UHMK1	U2AF homology motif (UHM) kinase 1 (UHMK1), mRNA.	2.85	0.00086	2.46	0.00043	2.66
Hs.430849	OSBPL8	Oxysterol binding protein-like 8 (OSBPL8), transcript variant 1, mRNA.	2.03	0.00022	3.05	0.00022	2.54
Hs.150557	KLF9	Basic transcription element binding protein 1 (BTEB1), mRNA.	2.6	0.00086	2.39	0.00043	2.50
Hs.210850	HECTD1	HECT domain containing 1 (HECTD1), mRNA.	2.35	0.00013	2.6	0.00086	2.47
Hs.142442	HP1BP3	Heterochromatin protein 1, binding protein 3	2.08	0.00086	2.41	0.00013	2.25
Hs.369284	ESF1	Chromosome 20 open reading frame 6 (C20orf6), mRNA.	2.13	0.00013	2.3	0.00086	2.21
Hs.119023	SMC2	SMC2 structural maintenance of chromosomes 2-like 1 (yeast) (SMC2L1), mRNA.	2.25	0.00022	2	0.00022	2.13
Hs.481927	NIPBL	Nipped-B homolog (Drosophila)	2.11	0.00043	2.04	0.00086	2.08
Hs.374201	KIF21A	kinesin family member 21A (KIF21A), mRNA.	2.13	0.00013	2	0.00013	2.06

Data includes genes with increased transcript levels displaying a ≥2-fold difference in average A vs. B and A vs. C.

### Functional associations of transcripts dysregulated in group A vs. control groups

In order to gain further understanding of the potential functional roles of dysregulated endometrial transcripts from group A, we obtained the functional annotations from each gene and determined the enriched processes associated to them from two different web-based tools. Within the down-regulated transcripts, the functional classifications *immune response* and *complement activation, classical pathway* were found to be statistically over-represented using the web based applications DAVID and GATHER respectively (p < 0.01). The Bayes factor obtained with the analysis using the GATHER database was 3, which indicates that the association of this particular function with the total of the transcripts in our gene list is weak. The up-regulated transcript list was not enriched with transcripts related to a particular function.

### P-regulated genes in women with implantation failure (group A) vs. control (group B)

We reasoned that the endometrium of women from group A might have a dysregulation in P-regulated transcripts as it has been described for endometriosis and also these genes might be coincident with those whose expression in the endometrium is altered upon treatment with the PR antagonist mifepristone. Since women from groups A and B only differ on the embryo implantation outcome, the list of dysregulated transcripts in group A vs. group B during the receptive phase of the endometrium was selected. Within this repertoire, we searched for those genes known to be regulated in normal cycling endometrium by P as it has been described before [4]. For that we accounted for those transcripts that, directed by P, get regulated for the acquisition of endometrial receptivity [24–32] and/or dysregulated in conditions that render the endometrium with an unreceptive phenotype (*i.e.*, endometriosis and mifepristone treatment) and that intersected with our list of up and down regulated genes (*i.e.*, A vs. B). We considered only those that had the opposite regulation compared with receptive endometrium, and same regulation in endometrium from women with compromised P signaling in the endometrium such as treated with mifepristone [7] and/or from women with endometriosis [4]. We found 14 and 86 up- and down-regulated genes respectively in the endometrium during the receptive period of women with implantation failure vs. control group B (Tables 5 and 6).

**Table 5:** **Genes previously described to be progesterone regulated that are down-regulated in endometrium of subjects with repeated embryo implantation failure**

UniGene ID	Gene symbol	Gene title	Up regulated in window of implantation	Down regulated in endometriosis or mifepristone	Fold change	p value
Hs.386793	GPX3	Glutathione peroxidase 3 (plasma) (GPX3), mRNA.	[25, 27, 29, 32]		0.01	0.00013
Hs.458355	C1S	Complement component 1, s subcomponent, transcript variant 1, mRNA.	[28, 29]		0.02	0.00043
Hs.647023	CLDN3	Claudin 3	[24]		0.07	0.00086
Hs.89603	MUC1	Mucin 1, transmembrane, mRNA.	[25]	[4]	0.11	0.00022
Hs.436657	CLU	Clusterin (complement lysis inhibitor, SP-40,40, sulfated glycoprotein 2, testosterone-repressed prostate message 2, apolipoprotein J), transcript variant 1, mRNA.	[27, 29, 31, 32]		0.12	0.00086
Hs.276770	CD52	CD52 molecule		[7]	0.14	0.00043
Hs.498173	SMPD1	Sphingomyelin phosphodiesterase 1, acid lysosomal (acid sphingomyelinase), transcript variant 1, mRNA.	[24, 26]		0.14	0.00013
Hs.523414	LOC492304	Putative insulin-like growth factor II associated protein, mRNA.	[27]		0.15	0.00022
**Hs.532325**	**PAEP**	**Progestagen-associated endometrial protein**	[24, 25, 27, 31, 32]	[39]	0.15	0.00086
Hs.590970	AXL	AXL receptor tyrosine kinase	[24]		0.15	0.00022
Hs.163893	PICALM	Phosphatidylinositol binding clathrin assembly protein		[4]	0.16	0.00086
Hs.525607	TNFAIP2	Tumor necrosis factor, alpha-induced protein 2, mRNA.	[27–29, 32]		0.18	0.00043
Hs.654439	APOE	Apolipoprotein E	[24, 29]		0.18	0.00086
Hs.201978	PTGS1	Prostaglandin-endoperoxide synthase 1 (prostaglandin G/H synthase and cyclooxygenase), transcript variant 2, mRNA.	[29]	[7]	0.19	0.00013
**Hs.82071**	**CITED2**	**Cbp/p300-interacting transactivator, with Glu/Asp-rich carboxy-terminal domain, 2, mRNA.**	[25]		0.19	0.00086
Hs.524518	STAT6	Signal transducer and activator of transcription 6, interleukin-4 induced, mRNA.	[29]		0.19	0.00022
Hs.478588	BCL6	B-cell CLL/lymphoma 6 (zinc finger protein 51), transcript variant 1, mRNA.	[25, 27, 29]		0.20	0.00086
**Hs.1012**	**C4BPA**	**Complement component 4 binding protein, alpha**	[24, 25, 27, 29, 31, 32]	[39]	0.22	0.00043
Hs.21765	FADS3	Fatty acid desaturase 3, mRNA.	[26]		0.23	0.00043
Hs.4055	KLF6	Kruppel-like factor 6	[25]		0.23	0.00013
Hs.332708	FBLN5	Fibulin 5, mRNA.	[27, 29]		0.23	0.00022
Hs.25292	JUNB	Jun B proto-oncogene, mRNA.	[25, 26]		0.25	0.00043
Hs.431048	ABL1	V-abl Abelson murine leukemia viral oncogene homolog 1, transcript variant b, mRNA.	[24]		0.27	0.00086
Hs.190783	HAL	Histidine ammonia-lyase	[26, 32]		0.27	0.00086
Hs.513984	FLII	Flightless I homolog (Drosophila), mRNA.	[24, 32]		0.27	0.00043
Hs.643357	ADAMTS1	ADAM metallopeptidase with thrombospondin type 1 motif, 1	[29]		0.29	0.00022
Hs.44227	HPSE	Heparanase	[29]		0.29	0.00086
Hs.515536	RRAS	Related RAS viral (r-ras) oncogene homolog, mRNA.	[27]		0.29	0.00013
Hs.409578	STK38	Serine/threonine kinase 38	[26]		0.29	0.00043
Hs.549171	C1orf56	Chromosome 1 open reading frame 56		[7]	0.29	0.00022
Hs.494457	NINJ1	Ninjurin 1, mRNA.	[26]		0.29	0.00013
Hs.270291	ACTN4	Actinin, alpha 4 (ACTN4), mRNA.	[29]		0.29	0.00086
Hs.381099	LCP1	Lymphocyte cytosolic protein 1 (L-plastin), mRNA.	[28, 29]		0.31	0.00043
Hs.185172	GNB2	Guanine nucleotide binding protein (G protein), beta polypeptide 2, mRNA.	[26]		0.31	0.00013
**Hs.1497**	**RARG**	**Retinoic acid receptor, gamma**		[7]	0.33	0.00043
Hs.474751	MYH9	Myosin, heavy polypeptide 9, non-muscle, mRNA.	[29]		0.33	0.00043
Hs.255093	PFKL	Phosphofructokinase, liver, transcript variant 2, mRNA.	[24]		0.33	0.00086
Hs.503911	NNMT	Nicotinamide N-methyltransferase	[25, 27]		0.33	0.00043
Hs.504877	ARHGDIB	Rho GDP dissociation inhibitor (GDI) beta , mRNA.	[24, 26, 27, 29]		0.33	0.00022
Hs.210995	CA12	Carbonic anhydrase XII, transcript variant 2, mRNA.	[27, 32]		0.35	0.00043
Hs.520640	ACTB	Actin, beta, mRNA.		[7]	0.35	0.00086
Hs.514819	AP2B1	Adaptor-related protein complex 2, beta 1 subunit, mRNA.	[24]		0.35	0.00013
Hs.511605	ANXA2	Annexin A2, transcript variant 2, mRNA.	[29]		0.35	0.00086
Hs.87752	MSN	Moesin, mRNA.	[29]		0.35	0.00022
Hs.654958	ABCF2	ATP-binding cassette, sub-family F (GCN20), member 2		[7]	0.35	0.00013
Hs.443577	TNFRSF21	Tumor necrosis factor receptor superfamily, member 21	[29]	[4]	0.35	0.00086
Hs.591868	ZBTB10	Zinc finger and BTB domain containing 10		[4]	0.35	0.00043
Hs.25348	VAMP2	Vesicle-associated membrane protein 2 (synaptobrevin 2)		[4]	0.38	0.00086
Hs.159161	ARHGDIA	Rho GDP dissociation inhibitor (GDI) alpha, mRNA.	[24]	[7]	0.38	0.00022
Hs.131269	RARRES1	Retinoic acid receptor responder (tazarotene induced) 1	[27]		0.38	0.00086
Hs.513915	CLDN7	Claudin 7, mRNA.	[25]		0.38	0.00013
Hs.10326	COPE	Coatomer protein complex, subunit epsilon, transcript variant 2, mRNA.	[24]		0.38	0.00013
Hs.416024	NRSN2	Neurensin 2		[7]	0.38	0.00043
Hs.434248	PLEC	Plectin	[26, 29]	[39]	0.38	0.00086
Hs.584854	AVIL	Advillin	[26, 29]		0.41	0.00022
Hs.183109	MAOA	Monoamine oxidase A	[24, 25, 27, 28, 31, 32]		0.41	0.00013
Hs.365405	SELO	Selenoprotein O		[4]	0.41	0.00013
Hs.645228	KIR3DL1	Killer cell immunoglobulin-like receptor, three domains, long cytoplasmic tail, 1	[29]		0.41	0.00086
Hs.528299	HTATIP	HIV-1 Tat interacting protein, 60 kDa, transcript variant 3, mRNA.	[26]		0.41	0.00043
Hs.164226	THBS1	Thrombospondin 1, mRNA.	[29]		0.41	0.00086
Hs.647078	CDK5	Cyclin-dependent kinase 5		[7]	0.41	0.00043
Hs.278573	CD59	CD59 antigen p18-20 (antigen identified by monoclonal antibodies 16.3A5, EJ16, EJ30, EL32 and G344), transcript variant 2, mRNA.	[29]		0.41	0.00022
Hs.515162	CALR	Calreticulin		[7]	0.41	0.00043
Hs.465744	INSR	Insulin receptor	[26]		0.41	0.00013
Hs.274256	ELOVL7	ELOVL family member 7, elongation of long chain fatty acids (yeast)		[4]	0.44	0.00086
Hs.450230	IGFBP3	Insulin-like growth factor binding protein 3	[27, 29, 32]		0.44	0.00086
Hs.504687	MYL9	Myosin, light polypeptide 9, regulatory	[27]		0.44	0.00022
Hs.446641	ARAF	V-raf murine sarcoma 3611 viral oncogene homolog, mRNA.	[25]		0.44	0.00086
Hs.2030	THBD	Thrombomodulin	[25, 27, 29]		0.44	0.00013
Hs.104672	FILIP1L	Filamin A interacting protein 1-like	[27]		0.44	0.00086
Hs.75862	SMAD4	SMAD family member 4		[4]	0.44	0.00022
Hs.520757	TBXAS1	Thromboxane A synthase 1 (platelet, cytochrome P450, family 5, subfamily A), transcript variant TXS-II, mRNA.	[29]		0.47	0.00013
Hs.283741	EXOSC5	Exosome component 5		[7]	0.47	0.00086
Hs.174312	TLR4	Toll-like receptor 4, transcript variant 2, mRNA.	[29]		0.47	0.00043
Hs.24601	FBLN1	Fibulin 1	[31]	[7]	0.47	0.00086
**Hs.501728**	**RHOG**	**Ras homolog gene family, member G (rho G)**		[7]	0.47	0.00043
Hs.220864	CHD2	Chromodomain helicase DNA binding protein 2		[4]	0.47	0.00086
Hs.524809	CLIP1	CAP-GLY domain containing linker protein 1	[29]		0.47	0.00043
Hs.92236	MLL4	Myeloid/lymphoid or mixed-lineage leukemia 4		[7]	0.47	0.00086
Hs.654688	MKL1	Megakaryoblastic leukemia (translocation) 1	[26]		0.47	0.00043
Hs.279837	GSTM2	Glutathione S-transferase mu 2 (muscle)	[26]		0.47	0.00086
Hs.645227	TGFB1	Transforming growth factor, beta 1		[7]	0.50	0.00086
Hs.149261	RUNX1	Runt-related transcription factor 1	[27]		0.50	0.00013
Hs.522818	L1CAM	L1 cell adhesion molecule (hydrocephalus, stenosis of aqueduct of Sylvius 1, MASA (mental retardation, aphasia, shuffling gait and adducted thumbs) syndrome, spastic paraplegia 1)	[26]		0.50	0.00043
Hs.840	IDO1	Indoleamine 2,3-dioxygenase 1	[24, 25, 29]		0.50	0.00086
Hs.2256	MMP7	Matrix metalloproteinase 7 (matrilysin, uterine)	[27]		0.50	0.00043

Data is expressed as fold change for endometrial genes down-regulated ≥2-fold in group A vs. group B that have been shown either up-regulated during the window of implantation or down-regulated in women with endometriosis or treated with mifepristone. Bolded transcripts are decreased also in group A vs. Group C.

**Table 6:** **Genes previously described to be progesterone regulated that are up-regulated in endometrium of subjects with repeated embryo implantation failure**

UniGene ID	Gene symbol	Gene title	Down regulated in window of implantation	Up regulated in endometriosis or RU486	Fold change	p value
Hs.208854	CD69	CD69 antigen (p60, early T-cell activation antigen)		[7]	2,3	0.00022
Hs.406515	NQO1	NAD(P)H dehydrogenase, quinone 1	[29]		2,2	0.00043
Hs.335614	SEC14L2	SEC14-like 2 (S. cerevisiae), mRNA.		[4]	1,9	0.00013
**Hs.481181**	**NEK1**	**NIMA (never in mitosis gene a)-related kinase 1 (NEK1), mRNA.**	[29]		1,6	0.00043
Hs.86368	CLGN	Calmegin, mRNA.		[7]	1,5	0.00086
**Hs.189075**	**TWF1**	**Twinfilin, actin-binding protein, homolog 1 (Drosophila)**		[4]	1,5	0.00043
Hs.127680	LOC389332	PREDICTED: hypothetical LOC389332 (LOC389332), mRNA.		[4]	1,4	0.00013
Hs.369430	PAM	Peptidylglycine alpha-amidating monooxygenase, transcript variant 3, mRNA.	[24, 28]		1,1	0.00022
**Hs.514806**	**GALNT1**	**UDP-N-acetyl-alpha-D-galactosamine:polypeptide N-acetylgalactosaminyltransferase 1 (GalNAc-T1), mRNA.**		[7]	1,1	0.00043
Hs.509447	GRLF1	Glucocorticoid receptor DNA binding factor 1		[7]	1,1	0.00086
**Hs.481927**	**NIPBL**	**Nipped-B homolog (Drosophila)**		[7]	1,1	0.00086
Hs.444558	KHDRBS3	KH domain containing, RNA binding, signal transduction associated 3, mRNA.	[24, 29, 32]	[4]	1,1	0.00043
Hs.495710	GPM6B	Glycoprotein M6B (GPM6B), transcript variant 4, mRNA.	[29]		1,0	0.00022
**Hs.496414**	**ATP7A**	**ATPase, Cu++ transporting, alpha polypeptide (Menkes syndrome)**	[29]		1,0	0.00043

Data is expressed as fold change for endometrial genes up-regulated ≥2-fold in group A vs. group B that have been shown either down-regulated during the window of implantation or up-regulated in women with endometriosis or treated with mifepristone. Bolded transcripts are increased also in group A vs. Group C.

### PROGINS detection

**Figure 2:**
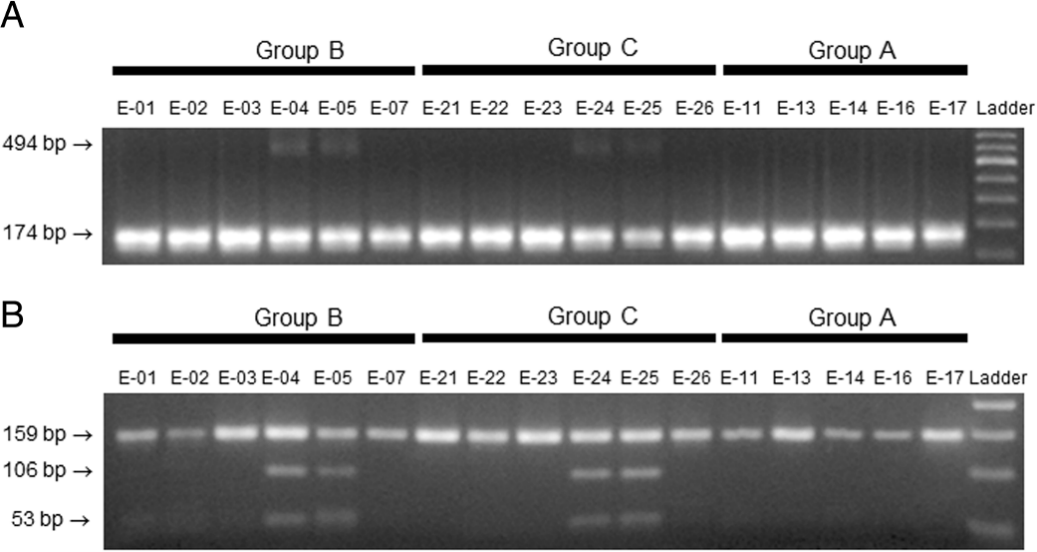
**Screening for PROGINS allele. A**, identification of Alu insertion in Intron G. The Alu insertion in the progesterone receptor gene generates a 494-bp PCR product compared to the 174-bp fragment obtained for the wild type. Samples 04, 05, 24 and 25 with bands at 494 bp and 174 bp indicate the presence of PROGINS in the heterozygous state. All the other lanes with a single fragment of 174 bp indicate the presence of the wild-type progesterone receptor in the homozygous state. **B**, restriction digestion of exon 5 with NlaIII. Lanes for samples 04, 05, 24 and 25 confirm the presence of PROGINS in heterozygous state; NlaIII cleaves the PCR product into two fragments, 106 and 53 bp. All the other samples displayed the uncleaved 159-bp fragment only, indicating the presence of the wild type receptor.

Since the comparative gene expression analysis of P-regulated genes in endometrial samples from group A, suggested an altered P response, we determined the presence of the Alu insertion in intron G of the PR gene (*PROGINS*) in women from groups A, B and C. Restriction fragment length polymorphism (RFLP) analysis was also carried out on exon 5 of PR gene for confirmation. We found 4 heterozygous subjects for *pgr* (Figures 2A and B). Two were from group B and two from group C, whereas no PROGINS alleles were detected in women from group A.

### IHC analysis

**Figure 3:**
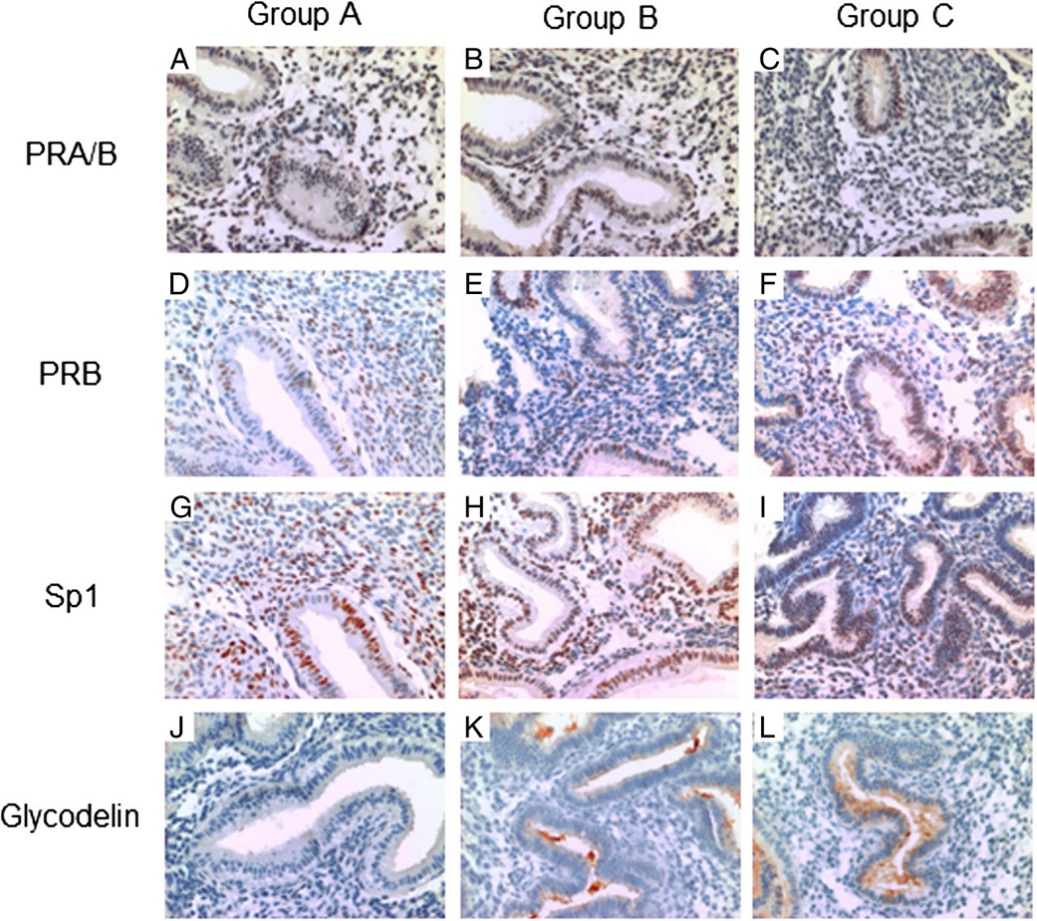
**Immunodetection of progesterone receptor (A and B isoforms, PR), progesterone receptor B (PRB), Specificity Protein 1 (Sp1) and glycodelin in endometrial sections.** Representative photomicrographs of endometrial sections immunostained in triplicate for PR (panels **A**, **B** and **C**), PRB (panels **D**, **E** and **F**), Sp1 (panels **G**, **H** and **I**) and glycodelin (panels **J**, **K** and **L**) are shown in women from group A (panels **A**, **D**, **G** and **J**; n = 5), group B (panels **B**, **E**, **H** and **K**; n = 6) and group C (panels **C**, **F**, **I** and **L**; n = 6).

**Figure 4:**
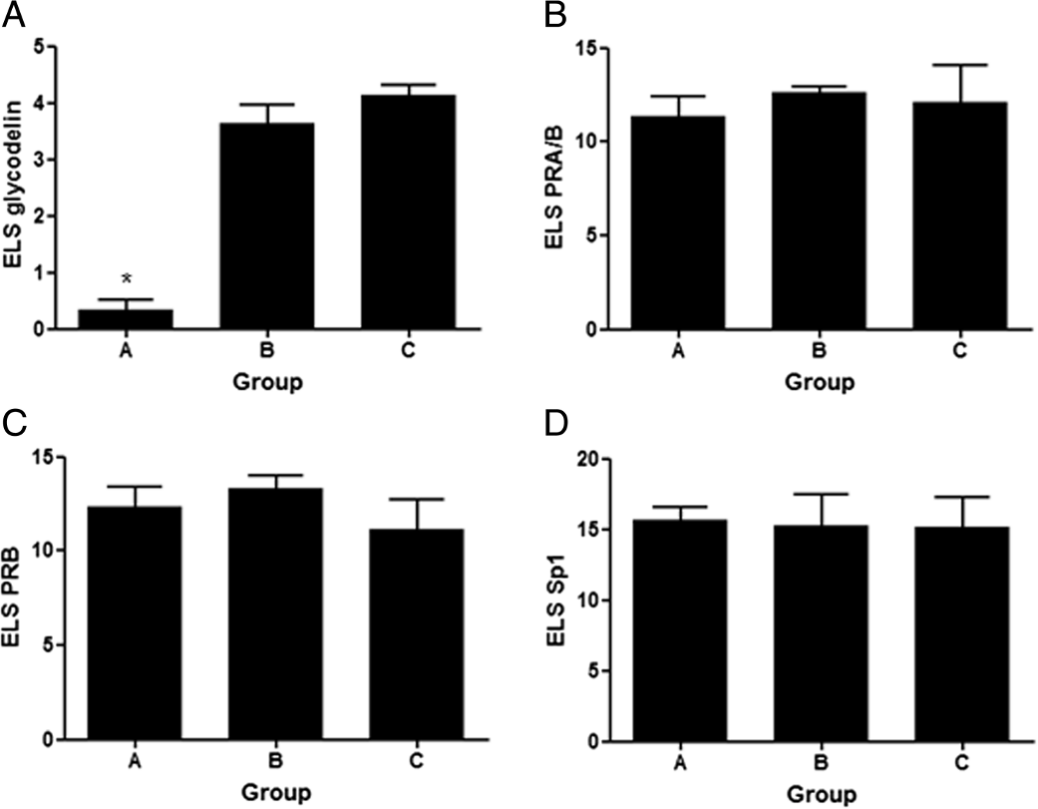
**Immunohistochemistry semiquantitation.** Expression Level Score (ELS) for immunostaining of glycodelin (panel **A**), PRA/B (panel **B**), PRB (panel **C**) and Sp1 (panel **D**) in endometrial sections from groups A (n = 5), B (n = 6) and C (n = 6). Data is expressed as average ELS ± SD for each group. *p < 0.05, Kruskal Wallis U-test.

Since the levels of both isoforms of PR in human endometrium have been found to be abnormal in patients with endometriosis [33, 34], we evaluated the immunoreactive presence of PR-A/B (Figures 3A and 3C), PR-B (Figures 3D and 3F) along with Sp1 (Figure 3G and 3I) and the P-regulated glycoprotein glycodelin (Figures 3 J and 3L) in paraformaldehyde-fixed paraffin embedded endometrial tissue from groups A, B and C by IHC. Immunostaining was semi-quantified by calculating the respective ELS scores for each detected molecule in all groups of women (Figure 4). ELS for glycodelin in groups B and C was 10.6 and 12.1 fold from group A respectively (p = 0.00509, Figure 4A). The presence of PR-A/B and PR-B in endometrial tissue was evaluated (Figures 3A-C and 3D-F, respectively), since a possible post-translational dysregulation of PR expression (not detected by transcript analysis) might explain the differential gene expression of P-regulated genes in the endometrium from women of group A such as glycodelin. The ELS scores obtained for PR-A/B and PRB did not show significant differences amongst groups (Figures 4B and 4C respectively). In addition, semi-quantitation of immunoreactive Sp1, a known co-activator and trans-activator of the PR that mediates P-induced glycodelin expression, did not show significant differences amongst groups A, B and C (Figure 4D).

## Discussion

Uterine receptivity is defined as a restricted time-related period when the uterus is receptive to blastocyst attachment and implantation. The establishment of this endometrial transition, which supports embryo implantation, is primarily coordinated by ovarian hormones, E2 and P that modulate uterine events in a spatiotemporal manner.

Endometrial factors, at the molecular level, have been suggested to explain some cases of infertility, recurrent miscarriages and implantation failure after IVF. In the present study we assessed the endometrial gene expression profile during the receptive period in mock oocyte donation cycles of women with repeated embryo implantation failure (Group A). Their profiles were compared with those obtained from women who achieved embryo implantation and pregnancy in oocyte donation cycles (Group B), or which got pregnant in natural spontaneous cycles (Group C). The data suggest a strong association between an aberrant endometrial gene expression and implantation failure. The stimulation protocol with steroid hormones performed before the endometrial sample collection was the same for all participating women in this study. Hence, the differential transcript profile in Group A suggests a long-term dysregulation of endometrial gene regulation rendering it not suitable for embryo implantation. The functional annotation analysis of dysregulated transcripts showed an enrichment of decreased genes involved in immune response and complement activation in women with repeated implantation failure.

Integration and cross-validation of endometrial transcripts regulated by P could increase the confidence in expression results for many more genes than is tractable with classical one-by-one validation of differentially expressed genes and should provide the up- and down-regulated genes that together orchestrate the acquisition of the receptive phenotype of the endometrium for embryo implantation. Such exploration and integration could help to get a comprehensive view of existing data needed to better prioritize experimental efforts. We identified a subset of P-regulated transcripts with differential expression in the endometrium of women from group A compared to the control group B revealing compromised P-signalling in the endometrium.

Pisarska *et al.* [23] reported that 42% of women with unexplained infertility carry the allele for the PROGINS mutation compared with 14% of control fertile women (with at least 1 term pregnancy). We analyzed the presence of the *PROGINS* allele in women from groups A, B and C and found no correlation between the *PROGINS* carrier women and altered transcript levels of P-regulated genes in the endometrium. This result is in line with a study from Coulam *et al.* [35] that did not find an association between PR polymorphisms with recurrent implantation failure in women after *in vitro* fertilization and embryo transfer.

Glycodelin, encoded by the gene *PAEP* [36, 37] is the main P-regulated glycoprotein secreted by the endometrial epithelium during the secretory phase and early pregnancy [38]. The transcript levels for *PAEP* have also been consistently identified to be one of the most abundant in the endometrium by several gene expression profiling studies [24, 25, 27, 31], and it has been shown to be decreased in women with endometriosis [39]. In the present study we found that the transcript levels for *PAEP* were decreased in the endometrium of women from group A compared to both control groups (Table 5). In addition, immunoreactive glycodelin evaluation in endometrial sections showed the protein to be significantly decreased in group A which is in line with the microarrays data. These results are consistent with the reduced concentrations of glycodelin in uterine flushing reported for patients with unexplained infertility [40]. In normal ovulatory cycles, P secretion is followed by endometrial glycodelin synthesis in epithelial glands from 4 to 5 postovulatory days onwards [37, 41]. Endometrial epithelial cells stimulated *in vitro* with progestins showed an increase in glycodelin transcription, synthesis, and secretion [42] however a PR-antagonist failed to prevent the induction of glycodelin [43]. *In silico* analysis of *PAEP* gene promoter sequence identified a potential P response element [44], however functional studies found that the transcription factor Sp1 mediates the effect of P and PR on human glycodelin expression in endometrial cells [45]. We semiquantified the immunoreactive levels of PR-A/B, PR-B and Sp1 in endometrial sections of women from groups A, B and C and found no significant differences between the groups.

The endometrial response to the increased circulating levels of P during the luteal phase has shown to be remarkably different in women with endometriosis compared to healthy controls. Such response has been evidenced by dysregulated specific gene networks of P-dependent genes in patients with endometriosis compared to non-diseased patients in eutopic secretory endometrium [4, 39, 46, 47]. This transcriptional behavior has led to the concept of ‘P resistance’ which may explain the association between pelvic endometriosis and infertility. We have found that the endometrial transcript profile from women with repeated implantation failure (group A) presents altered gene expression profile including several transcripts reported to be P-regulated, suggesting a women from group A have a compromised P signalling in the endometrium.

The cause of this endometrial defect is unknown, although the apparent intrinsic dysregulation in P signalling that renders the endometrium unreceptive in women with repeated embryo implantation failure seems to be beyond perturbations in PR expression such as chaperone proteins involved in receptor recycling and ligand binding [48], coregulators [49–51], as well as associated transcription factors and a variety of upstream signal transduction pathways capable of modifying PR and its coregulators [52–56]. In addition, the action of the P is not limited to the cell type in which is PR expressed since steroid hormone regulation can be mediated also through epithelial-stromal cross talk in the endometrium [57]. Also P can elicit a variety of rapid signalling events, independently of a direct transcriptional regulation or even in the absence of its cognate nuclear receptors [58] which may modulate gene expression. The molecular mechanism behind the defect in P-regulated gene networks in the endometrium of women with repeated embryo implantation failure is yet to be determined.

## Conclusions

We conclude that some cases of repeated implantation failure could be associated with an aberrant gene expression profile, particularly of transcripts related to the immune function and complement activation. Compromised P signaling might be the underlying mechanism for such endometrial gene expression deregulation in women with repeated implantation failure. Future research should focus on determining the causes of incomplete P signalling in the endometrium from these women.

## Abbreviations

DAVID: Database for annotation visualization and integrated discovery
E2: Estradiol
ELS: Expression level score
GATHER: Gene annotation tool to help explain relationships
IHC: Immunohistochemistry
mAdb: National cancer institute’s microarrays data base webtool
P: Progesterone
PAEP: Progestagen-associated endometrial protein
PCA: Principal component analysis
*Pgr*: Progesterone receptor gene
PR: Progesterone receptor
PR-A/B: Progesterone receptor isoforms A and B
PR-B: Progesterone receptor isoform B
PREs: Progesterone-response elements
PROGINS: Alu insertion in intron G of the progesterone receptor gene
RFLP: Restriction fragment length polymorphism
RT-PCR: Reverse transcription coupled to polymerase chain reaction
Sp1: Specificity Protein 1..
